# Time trends and social security burden of temporary work disability due to chronic venous disease in Brazil

**DOI:** 10.1186/s12889-020-08563-2

**Published:** 2020-04-10

**Authors:** Raissa M. da Coelho, Marco A. P. Nunes, Cristiane V. C. Gomes, Ilma S. dos Viana, Ângela M. da Silva

**Affiliations:** 1 Federal University of Sergipe Hospital , Rua Cláudio Batista, s/n, Bairro Cidade Nova, Aracaju- SE, CEP: 49060-108 Brazil; 2 National Institute of Social Security, Aracaju, Sergipe Brasil; 3 Postgraduate Program in Health Sciences, Federal University of Sergipe, São Cristóvão, Sergipe Brazil

**Keywords:** Chronic venous disease, Disability, Social security, Workers, Time series

## Abstract

**Background:**

Chronic venous disease (CVD) and disability are worldwide problems and have significant socioeconomic implications. This study aims to analyze the time trends and social security burden of temporary work disability due to CVD in Brazil.

**Methods:**

An ecological time series study using the Brazilian Social Security System database was performed from 2005 to 2014. Data from all benefits granted to workers with temporary disability due to CVD were analyzed. The cases were identified using diagnosis codes I83–I83.9 of the International Classification of Diseases 10th Revision (ICD-10). The time trend analyses were performed by the Joinpoint Regression Model, with sex, age, regions, income, and category of affiliation as variables. Crude and age-standardized rates were calculated.

**Results:**

A total of 429,438 benefits were granted for temporary work disability due to CVD from 2005 to 2014, with a growing trend and an age-standardized annual percent change (APC) of 3.4 (95% CI: 2.6–4.2) (*p* < 0.05). Social security expense increased 3.5-fold, and the number of days in benefit doubled from 2005 to 2014. In total, 27,017,818 working days were lost. The average duration of benefits was 55.3 days. The majority of workers were women (68.2%) (*p* < 0.001), between 30 and 59 years old, employed, had a monthly income ≤2 minimum wages (MW) (83.2%), and lived in the regions southeast (53.6%) and south (29.3%). Significantly higher APCs were observed for women than for men (APC: 4.9, 95% CI: 4.0–5.7 versus APC: 1.2, 95% CI: 0.1–2.4). All regions in Brazil had a significant growing trend, except in the north. No significant growth was observed in the age group of 60–69 years. A decreasing trend was observed in workers with monthly incomes above 2 MW (*p* < 0.05).

**Conclusions:**

Temporary work disability due to CVD and social security burden showed increasing trends with millions of working days lost, particularly among women and low-income workers. Preventing disability is challenging, and public policies are needed to reduce the social and economic impact of disability. Therefore, measures for promoting health at the workplace should be encouraged.

## Background

Chronic venous disease (CVD) is a worldwide problem and has high prevalence and significant socioeconomic implications [1–3]. Approximately half of the adult population has some form of venous disease [4, 5]. Varicose veins and venous ulcers are some of the clinical manifestations of CVD and are the main cause of chronic lower limb ulcers. In the United States, varicose veins affect over 25 million adults [4]. In Brazil, varicose veins are the main cause of absences from work, and CVD is one of the 15 diseases that significantly motivate the granting of temporary work disability benefits [6]. In European countries, CVD accounts for 1–2% of health care budgets [7].

It is estimated that 0.5–1.5% of the adult population will develop venous ulcers [8–10], which are found in advanced stages of CVD. Venous leg ulcers have delayed healing and high recurrence [10, 11]; therefore, this disease will limit various activities and result in temporary work absences and early retirement [6, 7, 10–12]. Although CVD is common in older adults, it also affects adults of working age and causes a significant loss in the number of work days [13, 14]. In addition to age, the risk factors for CVD included heredity, female sex, obesity, and number of pregnancies [4, 5, 8, 9, 15]. Furthermore, the relationship between work in a prolonged orthostatic position and CVD has been demonstrated [5, 15, 16].

As populations age around the world, the number of CVD cases is expected to increase. Similar trends have been observed for other age-associated, noncommunicable chronic diseases, and this will have direct effects on health and social security systems. In developing countries, these effects tend to be aggravated by the low incomes of large segments of the population and the inadequate access to health services [17, 18].

In Brazil, social security is an important source of morbidity data for the working population [19–21]. The National Institute of Social Security is the government agency that is responsible for granting pensions and disability benefits, including both work- and nonwork-related benefits. In 2014, 5.2 million benefits were granted, 44.7% of which were due to disability caused by diseases [22].

CVD is an underestimated public health problem, and few studies have addressed this issue in Brazil [10, 23]. Despite the evolution in the treatment of CVD in recent years, work disability due to CVD remains frequent, and the reasons are little known. The high prevalence of CVD, its significant socioeconomic impact [1–3], and the need to plan public policies aimed at preventing disabilities justify the initiation of this study. The objective of this study is to analyze the time trends and social security burden of temporary work disability due to CVD in Brazil between 2005 and 2014.

## Methods

### Data

Data from the Brazilian Social Security System database were used in this ecological time series study. The sample consisted of all benefits granted to workers with temporary disability due to CVD from 2004 to 2015. Each benefit granted for temporary work disability due to CVD in this period was considered a new case.

The International Classification of Diseases 10th Revision (ICD-10) [24] was used to identify the benefits granted because of CVD by using ICD-10 diagnosis codes I83–I83.9 (I83 [varicose veins of lower extremities], I83.0 [varicose veins of lower extremities with ulcer], I83.1 [varicose veins of lower extremities with inflammation], I83.2 [varicose veins of lower extremities with both ulcer and inflammation], and I83.9 [varicose veins of lower extremities without ulcer or inflammation]). These values were analyzed together.

The variables obtained were sex, age group (≤19, 20–24, 25–29, 30–34, 35–39, 40–44, 45–49, 50–54, 55–59, 60–64, 65–69, and ≥ 70 years), region (north, south, northeast, southeast, and midwest), affiliation category (employed, unemployed, special insured, self-employed, housekeeper, and others), year of concession (2005–2014), monthly expenses, and monthly income in units of minimum wage (MW). The payment of social security benefits is based on the MW set annually by the government. The current value of MW is approximately USD 250.

The period from January 1, 2005, to December 31, 2014, was used to determine the duration of benefits. To calculate the total lost working days among beneficiaries, an additional 15 days were added for each employee. During the period analyzed, it is possible that the same worker received more than one benefit. However, when the interval between two periods of disability due to the same illness is less than 60 days, the system considers the periods the same case.

The secondary code of ICD-10, which is used to identify cases of surgical convalescence and sociodemographic information (e.g., marital status and education level), was not included in the analysis because it is not available in the system database. Furthermore, beneficiaries aged ≤19 and ≥ 70 years were excluded from the time series analysis because they do not represent a significant portion of the economically active population.

### Social security

In Brazil, the majority of the working population is affiliated with the General Regime of Social Security (RGPS), which is governed by the National Institute of Social Security. All people over 16 can be affiliated with RGPS even if they do not have formal work relationships. For employed workers, the contribution is compulsory. Fishermen and farmers, who are categorized into the “special insured” group, make their contributions via their trade unions. The percentage of contribution varies according to the category of affiliation. The population covered by the RGPS may claim several benefits, such as for temporary work disability, pensions, and others. Categories with their own social security schemes, such as public and military officials, are not part of the RGPS.

The rules for granting benefits are provided in Brazilian law [25]. For the worker to be entitled to disability benefit, all employees with a minimum contribution period of 12 months to the RGPS are eligible for the benefit. After the grace period has been fulfilled, the maintenance of the condition of the insured person will last for 12 months even in case of job loss. For workers who have 120 contributions or more, this condition is extended for another 12 months. The grace period is waived only in case of accidents and for serious illnesses indicated in the law [25].

In addition to RGPS affiliation and contributions, an assessment by an expert physician is mandatory to obtain temporary work disability benefits. Expert physicians check information such as the date on which the illness started, the date on which the disability began, the disease diagnosis based on ICD-10, and the period of receipt of the benefit.

According to Brazilian law, companies are responsible for paying the first 15 days of work disability of employees. However, there is no database available to query the causes of disability for this period. The information provided by the Brazilian Social Security System refers to periods of disability exceeding 15 days and the reason for the granting of the benefit. For other workers, the entire period is covered by the Brazilian Social Security System. The military and some public servants have their own social security regimes.

### Statistical analyses

The frequency of temporary work disability due to CVD was determined by analyzing the time trends. The reference population was determined by the Brazilian Institute of Geography and Statistics. The total number of workers with employment contracts was not used because data from the other categories of affiliation, which include self-employed, unemployed, fishermen, rural workers, and others, were also included in the analyses. Crude rates, age-specific rates, and age-adjusted rates adjusted by world population [26, 27] were calculated. The 95% confidence intervals (95% CI) were calculated using the formula 95% CI = R +/− (1.96 × SE), where R is the annual index, and SE is a standard error. The standard error was calculated using the formula SE = R / √N, where R is the annual index, and N is the number of cases per year.

The Joinpoint Regression Program (version 4.1.1.5) [28] was used to calculate the time trends for the consecutive 10-year series of temporary work disability due to CVD. Crude and age-standardized rates were used as dependent variables, and the year was used as the independent variable. The analysis started with zero joinpoints (a straight line) and tested whether additional joinpoints need to be added to the model. To test the significance, the program uses the Monte Carlo permutation test [29] to find the best fit and to determine an annual percent change (APC) for each line segment. The 95% confidence intervals (95% CIs) were calculated for each estimated APC. If these intervals excluded zero, then the APCs were statistically significant (*p* < 0.05).

## Results

In Brazil, 429,438 benefits were granted for temporary work disability due to CVD from 2005 to 2014, and a growing trend of work disability due to CVD was observed in women and low-income workers. The social security expense within the first 30 days of benefits due to CVD increased 3.5-fold from BRL 14.9 in 2005 to BRL 52.1 million in 2014. The number of days of benefits per year doubled from 1,369,709 in 2005 to 2,810,151 in 2014. In total, 27,017,818 workdays were lost, including 23,593,453 days covered by the social security system and 3,424,365 covered by company payments for the first 15 days of employee disability. The average duration of benefits was 55.3 days (standard deviation [SD]: 40.8 days).

Table 1 shows the characteristics of workers with temporary work disability due to CVD in Brazil from 2005 to 2014. Most of the workers were female (68.2%). There were significant differences between genders (*p* < 0.001), except for the ≤19-year-old age group. Approximately 85.6% of workers were between 30 and 59 years old. The percentage of workers employed and earning more than 2 MW was higher among men. Self-employed individuals, housekeepers, and special insured persons were predominantly women. Approximately half of the benefits were granted in the southeast region (53.6%), followed by the benefits in the south region (29.3%).

**Table 1:** Characteristics of workers with temporary work disability due to CVD, Brazil, 2005–2014

Variables	Female (***n*** = 292,800)	Male (***n*** = 136,638)	***P*** value**
	n (%)	n (%)	
Age group			
< 19	1219 (0.4)	541 (0.4)	0.343
20–29	22,747 (7.8)	13,910 (10.2)	< 0.001
30–39	75,901 (25.9)	34,602 (25.3)	< 0.001
40–49	99,648 (34.0)	44,338 (32.5)	< 0.001
50–59	78,202 (26.7)	34,954 (25.6)	< 0.001
60–69	14,341 (4.9)	8171 (6.0)	< 0.001
> 70	742 (0.3)	122 (0.1)	< 0.001
Affiliation category			
Employee	139,641 (47.7)	88,650 (64.9)	< 0.001
Self employed	65,667 (22.4)	19,378 (14.2)	< 0.001
Housekeeper	29,065 (9.9)	667 (4.9)	< 0.001
Special*	27,764 (9.5)	9971 (7.3)	< 0.001
Unemployed	17,978 (6.1)	15,817 (11.6)	< 0.001
Others	12,685 (4.3)	2155 (1.6)	< 0.001
Income (monthly)			
≤ 2 MW	265,964 (90.8)	91,439 (66.9)	< 0.001
2–4 MW	22,522 (7.7)	35,354 (25.9)	< 0.001
≥ 4 MW	4314 (1.5)	9845 (7.2)	< 0.001
Regions			
Southeast	154,141 (52.6)	76,079 (55.7)	< 0.001
South	88,705 (30.3)	37,126 (27.2)	< 0.001
Northeast	26,054 (8.9)	13,645 (10.0)	< 0.001
Midwest	21,007 (7.2)	7828 (5.7)	< 0.001
North	2893 (1.0)	1960 (1.4)	< 0.001

*Special, rural workers and fishermen; *CVD* Chronic venous disease, *MW* minimum wage

** Chi-Square Test

**Fig. 1:**
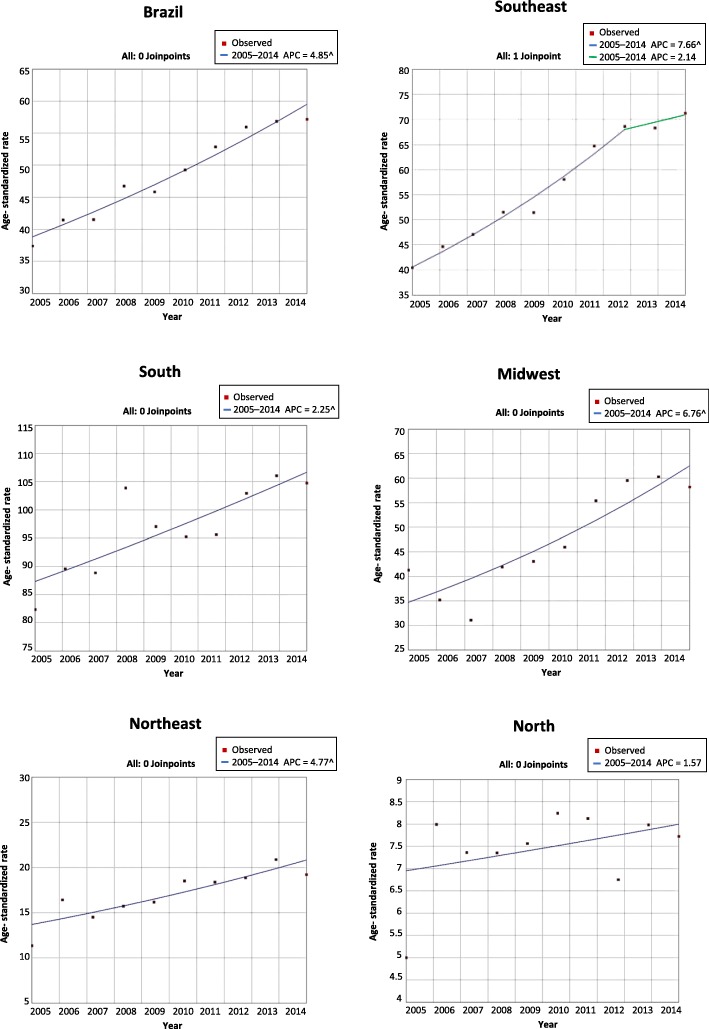
Temporal trends of work disability due to CVD for female and regions, Brazil, 2005–2014

In the period studied, an increasing trend was observed in cases of temporary work disability due to CVD in Brazil, with crude and age-standardized APC values of 3.9 (95% CI: 3.1–3.7) and 3.4 (95% CI: 2.6–4.2), respectively. Table 2 shows the crude and age-standardized rates of work disability due to CVD by sex and region. The age-standardized rate analysis did not show significant growth in the north region. In Brazil, a significantly higher APC was observed for women than for men (APC: 4.9, 95% CI: 4.0–5.7 versus APC: 1.2, 95% CI: 0.1–2.4) (*p* < 0.05). Among women in the southeast region, there was a change in the growth trend in the granting of benefits. From 2005 to 2012, the crude and age-standardized APC values increased to 8.8 (95% CI: 7.1–10.4) and 7.7 (95% CI: 6.0–9.4), respectively. By contrast, no significant growth was observed from 2012 to 2014 (Table 2 and Fig. 1).

**Table 2:** Temporal trends of work disability due to CVD by sex and regions, Brazil, 2005–2014

	Segment	Period	Crude rates		Age-standardized rates	
			APC	95% CI	APC	95% CI
Brazil	1	2005–2014	3.9^	3.1 to 4.7	3.4^	2.6 to 4.2
Southeast	1	2005–2014	5.2^	4.3 to 6.0	4.6^	3.8 to 5.5
Midwest	1	2005–2014	6.0^	3.2 to 8.8	5.3^	2.6 to 8.0
Northeast	1	2005–2014	4.5^	2.5 to 6.6	3.6^	1.6 to 5.6
South	1	2005–2014	1.5^	0.4 to 2.6	1.3^	0.2 to 2.4
North	1	2005–2014	1.5	−0.7 to 3.8	0.6	−1.4 to 2.8
Female						
Brazil	1	2005–2014	6.1^	5.2 to 7.0	4.9^	4.0 to 5.7
Southeast	1	2005–2012	8.8^	7.1 to 10.4	7.7^	6.0 to 9.4
	2	2012–2014	2.8	−6.3 to 12.8	2.1	−5.3 to 10.2
Midwest	1	2005–2014	8.2^	5.2 to 11.2	6.8^	3.9 to 9.7
Northeast	1	2005–2014	6.6^	4.3 to 9.0	4.8^	2.5 to 7.1
South	1	2005–2014	3.0^	1.7 to 4.3	2.2^	1.0 to 3.5
North	1	2005–2014	3.7^	0.5 to 7.1	1.6	−1.5 to 4.7
Male						
Brazil	1	2005–2014	2.4^	1.3 to 3.6	1.2^	0.1 to 2.4
Southeast	1	2005–2014	3.1^	2.0 to 4.2	2.1^	1.0 to 3.2
Midwest	1	2005–2014	4.0^	1.6 to 6.4	2.6^	0.2 to 5.1
Northeast	1	2005–2014	4.2^	1.5 to 6.9	2.3	−0.2 to 4.9
South	1	2005–2014	0.3	−0.8 to 1.4	−0.6	−1.7 to 0.5
North	1	2005–2014	2.2	− 1.2 to 5.7	0.7	−2.8 to 4.2

*CVD* Chronic venous disease, *APC* Annual percentage change; *CI* Confidence interval

^ APC *p < 0.05*

**Fig. 2:**
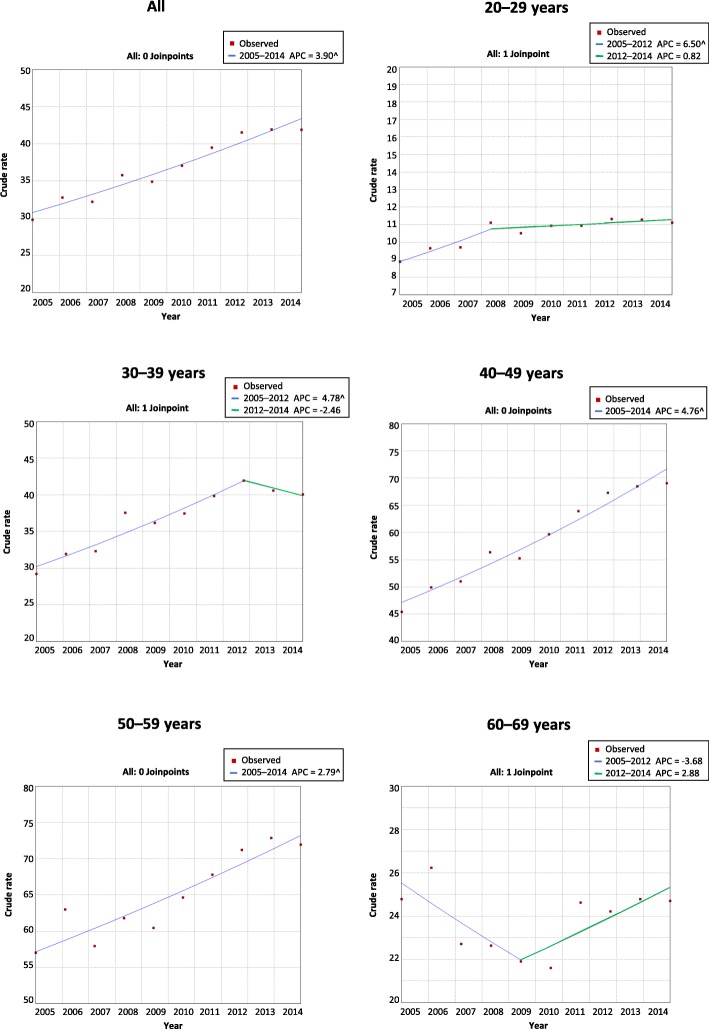
Temporal trends of work disability due to CVD by age group, Brazil, 2005–2014

Figure 2 and Table 3 show the temporal trends of work disability due to CVD by age group and regions in Brazil. Increasing trends were most notable in the southeast and midwest regions. From 2005 to 2008, workers aged 20–29 years in the southeast region had the highest APC (APC: 8.9; 95% CI: 2.7–15.5). From 2005 to 2014, workers aged 30–39 years (APC: 6.5; 95% CI: 3.1–10.0) and 40–49 years (APC: 6.7; 95% CI: 3.8–9.6) from the midwest region had the highest APCs. In the south region, there was an increase only in the group aged 40–49 years (APC: 3.1; 95% CI: 1.9–4.3). In the 60- to 69-year-old age group, there was no significant growth in any region. We showed that there was a downward trend from 2005 to 2009, followed by growth from 2009 to 2014, without significant APC (Fig. 2 and Table 3).

**Table 3:** Temporal trends of work disability due to CVD by age group and regions, Brazil, 2005–2014

	Segment	Period	Crude rates	
			APC	95% CI
Brazil				
20–29	1	2005–2008	6.5^	2.2 to 11.0
	2	2008–2014	0.8	− 0.5 to 2.1
30–39	1	2005–2012	4.8^	2.7 to 6.9
	2	2012–2014	−2.5	− 14.3 to 11.1
40–49	1	2005–2014	4.8^	4.0 to 5.6
50–59	1	2005–2014	2.8^	1.8 to 3.7
60–69	1	2005–2009	−3.7	−9.4 to 2.4
	2	2009–2014	2.9	−1.0 to 6.9
Southeast				
20–29	1	2005–2008	8.9^	2.7 to 15.5
	2	2008–2014	1.4	−0.4 to 3.3
30–39	1	2005–2012	7.3^	5.3 to 9.2
	2	2012–2014	−2.1	−12.8 to 9.9
40–49	1	2005–2014	6.3^	5.3 to 7.3
50–59	1	2005–2014	3.9^	3.0 to 4.8
60–69	1	2005–2009	−5.3	−10.5 to 0.3
	2	2009–2014	2.9	−0.8 to 6.8
Midwest				
20–29	1	2005–2014	4.1^	1.7 to 6.5
30–39	1	2005–2014	6.5^	3.1 to 10.0
40–49	1	2005–2014	6.7^	3.8 to 9.6
50–59	1	2005–2014	4.8^	2.1 to 7.5
60–69	1	2005–2007	−13.8	−37.1 to 18.2
	2	2007–2014	2.0	−2.0 to 6.1
Northeast				
20–29	1	2005–2014	5.5^	2.0 to 9.3
30–39	1	2005–2007	25.8	−7.0 to 70.1
	2	2007–2014	2.9	−0.1 to 6.0
40–49	1	2005–2014	5.0^	2.6 to 7.4
50–59	1	2005–2014	1.5	−0.4 to 3.4
60–69	1	2005–2014	0.5	−1.5 to 2.5
South				
20–29	1	2005–2014	−0.7	−2.2 to 0.8
30–39	1	2005–2014	0.1	−1.4 to 1.6
40–49	1	2005–2014	3.1^	1.9 to 4.3
50–59	1	2005–2014	1.0	−0.1 to 2.1
60–69	1	2005–2014	1.1	−0.4 to 2.6
North				
20–29	1	2005–2014	0.2	−4.7 to 5.4
30–39	1	2005–2008	21.8	−5.4 to 56.8
	2	2008–2014	−2.7	−8.6 to 3.5
40–49	1	2005–2014	1.0	−0.9 to 2.9
50–59	1	2005–2014	−0.8	−3.9 to 2.4
60–69	1	2005–2014	1.4	−3.7 to 6.7

*CVD* Chronic venous disease, *APC* Annual percentage change;

*CI* confidence interval; ^ APC *p < 0.05*

The analysis by affiliation category showed a growing trend for employed workers from 2005 to 2012 and for self-employed workers from 2005 to 2014. Special insured workers had a significant decrease (*p* < 0.05). We identified an increasing trend of temporary work disability due to CVD in workers with monthly income ≤2 MW, with a crude and age-standardized APC of 5.1 (95% CI: 3.7–6.5) and 4.1 (95% CI: 2.7–5.6), respectively. A significantly decreasing trend was observed in workers with a monthly income above 2 MW (*p* < 0.05).

## Discussion

This study demonstrated the dimension of work disability due to CVD in Brazil. CVD has caused the loss of millions of working days in developed countries [11–14], and temporary work disability due to CVD and social security burden have shown increasing trends. This finding can be attributed to several factors: the high prevalence of the disease in the adult population [4, 5, 9], the greater participation of women in the labor market [6], and the increasing prevalence of chronic diseases (e.g., obesity [30], which is one of the recognized risk factors for CVD) [15, 31].

In agreement with the literature, it was found that women were the most affected by CVD [4, 5, 9, 23]. In addition to obesity, prolonged orthostatic work and number of pregnancies are also risk factors for CVD [4, 5, 8, 9, 16, 23]. Although a small change in the trend was observed in the southeast region from 2012 to 2014, particularly among female workers aged 20–29 years old and 30–39 years old, it was not possible to define the reasons for this change.

In more developed regions, such as the southeast region, it is possible that minimally invasive therapies for CVD are more accessible, thus allowing workers to return to work earlier [32, 33]. The majority of the population in Brazil has a low income and is dependent on the public health system, in which specialized treatment is limited. Therefore, the possible inequalities of access to appropriate treatment must be considered.

There were increasing and decreasing trends of work disability due to CVD in workers with monthly income ≤2 MW and in workers with higher incomes, respectively. Regarding chronic ulcers of the lower limbs, which occur in the most advanced stage of CVD, studies conducted in Brazil have shown that the disease was more prevalent in the low-income population [10, 34]. This finding is similar to those of studies performed in the United Kingdom [35, 36].

Although CVD is more prevalent in the elderly, we found that the majority of workers with CVD disability were less than 60 years old. One study found a higher prevalence of varicose veins among young people in Brazil compared with other countries, thus suggesting that CVD could appear in the earlier stages of life [9]. More severe forms of CVD, such as venous ulcers, are also more prevalent in the elderly; however, there was no significant increase in beneficiaries in the age group of 60–69 years old. One of the reasons for this finding could be the healthy worker effect because the probability that a healthy worker will be employed is greater than the probability that an unhealthy worker will be employed [37]. In Brazil, the low minimum age of retirement may also influence these data. Most pensions are granted only on the basis of contribution time with no minimum age requirement. This has resulted in a population of retirees under 60.

Owing to an aging population and changes to retirement rules, the number of people with temporary work disability due to CVD has increased; this situation has led to an increase in the burden on social security, the health care system, and employers. Disability due to CVD also affects companies because they pay for the first 15 days of work leave and lose productivity. Some studies conducted from the social security database have only used information from workers affiliated as employees [19–21]. However, in the current study, we present data from all workers who received temporary work disability benefit due to CVD regardless of affiliation category. This resulted in the greater reliability of data because approximately half of the workers would be excluded if we considered only the employees.

Brazil is a country with an expansive territory and significant social inequalities. Thus, we analyzed the granting of benefits by region and found a predominance of concessions in the southeast region. One reason for this finding is that the southeast region is the most populous and industrialized region of Brazil and has the largest number of workers with social security coverage. By contrast, the north region had the lowest amount of benefits granted for CVD and is the region with the lowest social security coverage [22].

Furthermore, an extensive part of the north region of Brazil is covered by the Amazon rainforest, and the only means of transportation in some places is by boat. Although there are boats with social security services, communities located in more isolated areas in the north would still have to travel long distances, thus making it difficult to access these services. For people with low incomes, the cost of transportation may be a limiting factor. The benefit claim can be made over the internet or by telephone, but medical evaluation for the initial grant of the benefit needs to be performed in person.

This study has some limitations. The time series analysis is based on the secondary population data of workers affiliated with the RGPS, which does not represent all Brazilian workers. Given that this is an ecological study, individual characteristics and risk factors were not analyzed. Furthermore, the number of benefits granted might not accurately represent the number of workers because it was not possible to identify whether the same worker received more than one benefit due to CVD during the analyzed period. Despite the expressive number of benefits granted due to CVD, the rate of work disability due to CVD may be underestimated because the effect of the healthy worker and because of the emergence of minimally invasive therapies for CVD with less recovery time [32, 33, 37]. However, this study used official data from the largest database of the Brazilian working population.

## Conclusions

This time series study identified the increasing trends of work disability and social security burden due to CVD in Brazil. The results showed that CVD has led to the loss of millions of working days, particularly among women. Owing to the increase in life expectancy and retirement age, it is possible that more people will develop work disability. Preventing disability is challenging, and public policies are needed to reduce the social and economic impact of disability.

We believe that this study may contribute to the planning of public policies aimed at preventing disabilities. Although many factors are related to the development and progression of CVD, strategies that are focused on modifiable risk factors such as obesity and prolonged standing should be encouraged in the workplace. Furthermore, identifying workers with CVD and instituting appropriate treatment early can reduce the disability caused by CVD and the burden on social security. Future research is needed to evaluate measures that could modify the trend presented in this study.

## Data Availability

The data that support the findings of this study were obtained from the Citizen Information Service (https://esic.cgu.gov.br/sistema/site/index.aspx).

## Abbreviations

APC: Annual percent change
CI: Confidence interval
CVD: Chronic venous disease
ICD-10: International classification of diseases 10th revision
MW: Minimum wage
RGPS: General regime of social security
SD: Standard deviation
